# Clinical Analysis of Adult Severe Open-Globe Injuries in Central China

**DOI:** 10.3389/fmed.2021.755158

**Published:** 2021-10-27

**Authors:** Hongling Chen, Junjun Han, Xianliang Zhang, Xuemin Jin

**Affiliations:** ^1^Department of Ophthalmology, Henan Eye Institute, Henan Eye Hospital, Henan Provincial People's Hospital, People's Hospital of Zhengzhou University, Zhengzhou, China; ^2^Department of Ophthalmology, The First Affiliated Hospital of Zhengzhou University, Zhengzhou, China

**Keywords:** adult, central China, open-globe injuries, trauma, epidemiology

## Abstract

**Purpose:** To describe the characteristics, management, and outcomes of adult severe open-globe injured (OGI) eyes.

**Methods:** Retrospective chart review of inpatients with initial visual acuity (VA) of light perception (LP) or no light perception (NLP) associated with OGI between 2017 and 2020 at Department of Ophthalmology, Henan Eye Institute, Henan Eye Hospital, Henan provincial People's Hospital.

**Results:** Six hundred twenty-five eyes of 622 adult patients with initial VA of LP or NLP associated with open-globe injuries (OGIs) were included. The mean age was 47.8 ± 14.1 years with the range from 18 to 91 years. Significant male predominance was noted (81.5%). The most common type of these severe OGIs was rupture (65.8%). Traffic accidents accounted for 13.5% followed by fall/tumble (10.9%) and nail/wire (10.9%) of all the severe OGIs. Almost half of the injuries happened at workplace (47.2%). Initially, 78.7% eyes just received primary debridement and wound closure, while 8.5% eyes with no possible of anatomical reconstruction received evisceration. After initial management, 350 eyes received subsequent operation, including 239 eyes underwent vitrectomy + silicone oil/(+cataract remove). Finally, over 6 months follow-up, 137 eyes (21.9%) were eviscerated, 150 eyes (24.0%) got atrophied, while 132 eyes (21.1%) retain some VA. Fifty-three eyes (8.5%) got VA of 0.3–1.5.

**Conclusion:** Severe OGIs are most seen in the young, middle-aged, and male working population and remain a serious public health problem, resulting in significant vision loss or Evisceration of eyes. Effective preventive measures should be taken for the individuals in these groups.

## Introduction

Open-globe injuries (OGIs) include a full-thickness break or rupture of the cornea and/or the sclera. OGIs are the major cause of unilateral visual loss. Severe OGIs (initial VA of LP or NLP) not only lead to vision loss but also can cause eye loss. OGIs seem more common in developing countries than in developed countries (1). Henan province is a large agricultural province. It is located in central China with rich labor resources. Latest population census shows that the population of Henan was about one hundred million (99,366 thousands). Henan Eye Hospital is the 1st or the second largest ophthalmic center in Henan Province and received quite a lot of ocular trauma patients every year in Henan Province.

As we know, OGIs are most seen in working population and remain a serious public health problem, resulting in significant vision loss. In severe OGIs, eyes had been destroyed or cannot be reconstructed. A good understanding of the characteristics of severe OGIs in adults is required for the determination of preventive measures. In this study, we aimed to separately analyze the characteristics of adult severe OGIs diagnosed and treated at Department of Ophthalmology, Henan Eye Institute, Henan Eye Hospital, Henan provincial People's Hospital.

## Methods

We retrospectively chart reviewed all the patients diagnosed and treated at Department of Ophthalmology, Henan Eye Institute, Henan Eye Hospital, Henan provincial People's Hospital between 2017 and 2020. The OGI patients aged over 18 and with initial VA of LP or NLP were included into the study. Age, gender, mechanism of injury, surgical options, and outcomes were analyzed. Ethics approval for the study was granted by Henan Eye Institute, Henan Eye Hospital, Henan provincial People's Hospital Human Research Ethics Committee. The study adhered to the tenets of the Declaration of Helsinki.

### Classification

Open-globe injuries were classified according to the Birmingham Eye Trauma Terminology System (2).

The patients were classified into 7 age groups: 18–29, 30–39, 40–49, 50–59, 60–69, 70–79, ≥80 years.

### Statistical Analysis

Data were analyzed using Microsoft Office Excel 2007. Continuous and categorical variables were displayed as means ± standard deviation (SD) and percentages, respectively.

The ethics number is HNEECKY-2021(33).

## Results

**Figure 1:**
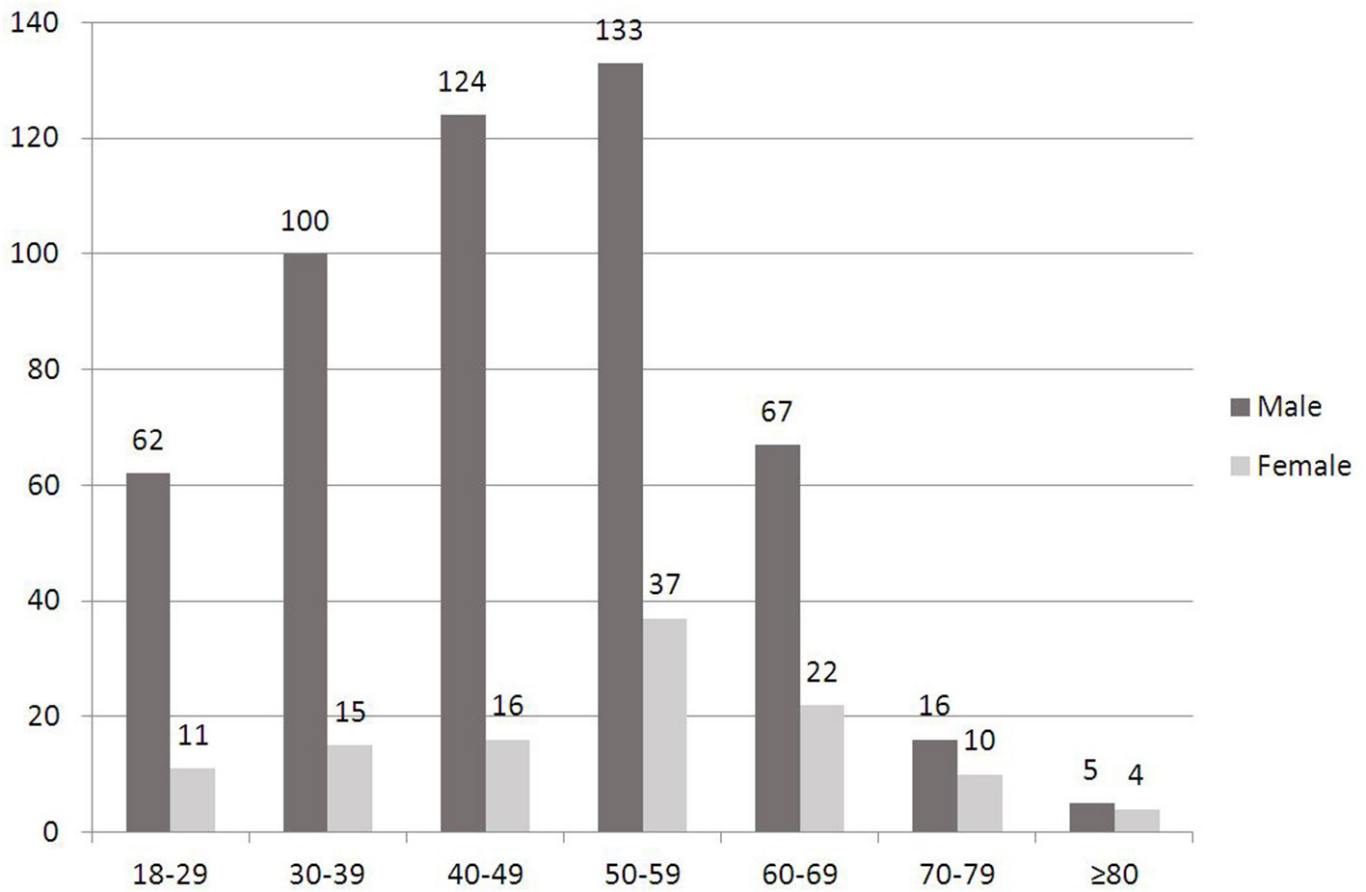
Distribution of gender and age groups of severe adult opened globe injury patients.

In the 4 years, 1,902 cases (1,908 eyes) of OGI patients (including 1,483 eyes of 1,478 patients older than 17 years) were diagnosed and treated at Department of Ophthalmology, Henan Eye Institute, Henan Eye Hospital, Henan provincial People's Hospital. Of all the OGIs, 622 cases (625 eyes) of adult severe OGIs with initial VA of LP or NLP were included in this study. The mean age at the time of injury was 47.8 ± 14.1 years with the range from 18 to 91 years. Significant male predominance was noted (81.5%). The highest incidence of severe adult OGIs was found in the middle-aged group (50–59, 40–49, 30–39) in male patients (Figure 1). The highest incidence of severe adult OGIs was found in 50–59 age group in female patients. Of the 622 patients, 3 cases had bilateral eye injury. In patients with unilateral eye injury, 299 (48.2%) eyes were right eye and 320 (51.2%) were left eye.

**Figure 2:**
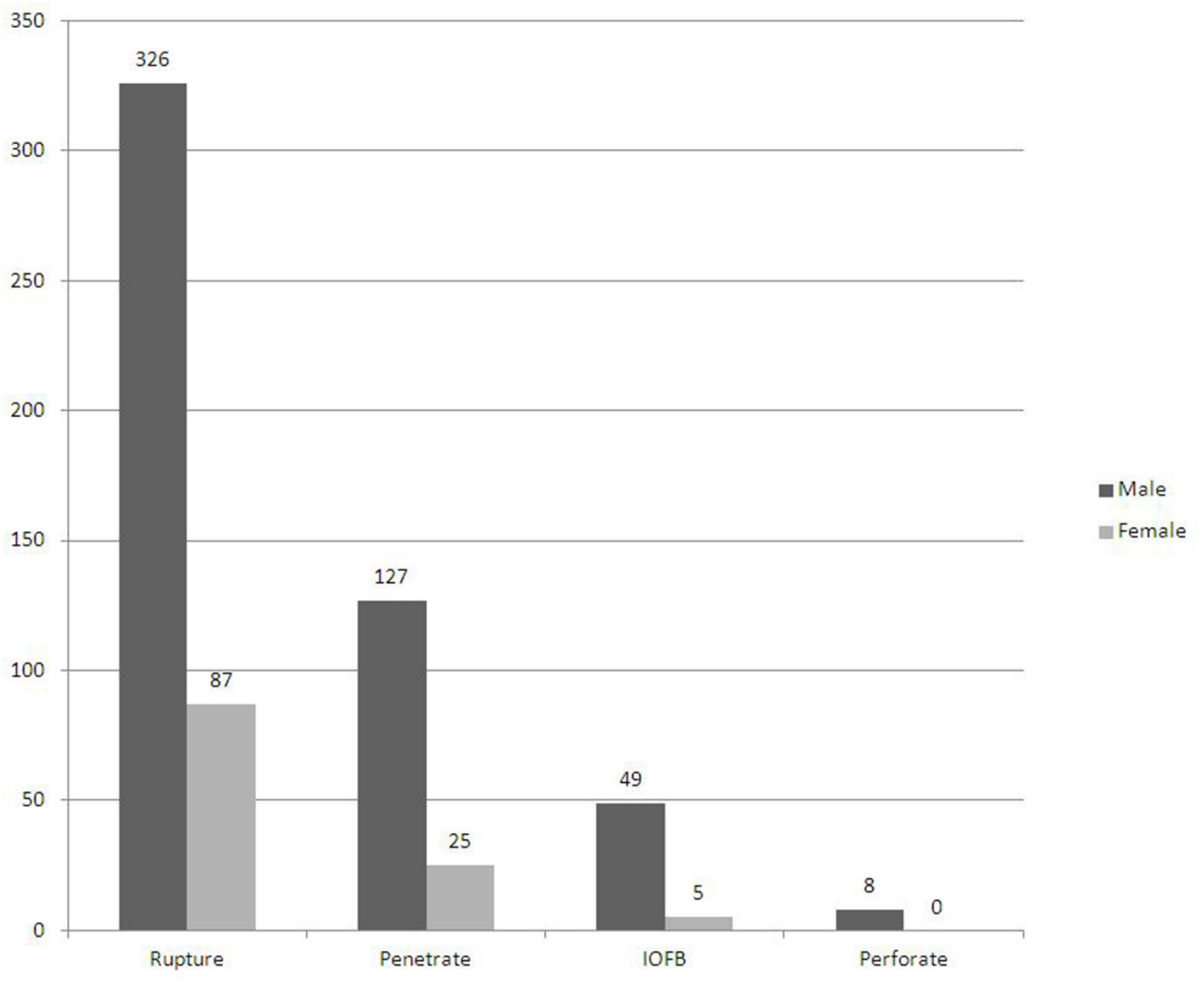
Distribution of gender and types of opened globe injury patients.

In the present study, the most common type of severe OGIs was rupture (65.8%) no matter in male (326 eyes) or female (87 eyes) (Figure 2). Traffic accidents accounted for 13.5% followed by fall/tumble (10.9%) and nail/wire (10.9%) of all the severe OGIs (Table 1). Almost half of the injuries happened at workplace (47.2%).

**Table 1:** Causes of severe open globe injuries.

**Cause**	***n***	**%**
Traffic accident	84	13.5
Fall, tumble	68	10.9
Nail, wire	68	10.9
Wood, branch, bamboo	58	9.3
Fireworks, firecrackers	43	6.9
Emery cutter, grinding wheel, electric saw	37	6
Violence	34	5.5
Metal bar	30	4.8
Metal fragments caused by hammering	23	3.7
Metal block, sheet metal	21	3.4
Scissors, knife	12	1.9
Flying stone	12	1.9
Lighter, bottle, bulb explosion	7	1.1
Glass	5	0.8
Falling objects	4	0.6
Metal hook	3	0.5
Toy bullet	3	0.5
Children head	2	0.3
Battery explosion	2	0.3
Flying object from grass trimmer	1	0.2
Water pump explosion	1	0.2
Tire explosion	1	0.2
Pressure cooker explosion	1	0.2
Gas tank explosion	1	0.2
Cock peck	1	0.2
Horn	1	0.2
Others and unknown	99	15.9
Total	622	100

Initially, 492 eyes (78.7%) received primary debridement and wound closure. Fifty three eyes (8.5%) with no possible of anatomical reconstruction received evisceration (Table 2). After initial management, 350 eyes received subsequent operation, including 239 eyes underwent vitrectomy + silicone oil/ (+cataract remove) and 58 eyes got eviscerated (Table 3). Over 6 months of follow-up, 137 eyes (21.9%) were eviscerated, 150 eyes (24.0%) got atrophied, and 132 eyes (21.1%) retain some VA. At the end of follow up, fifty-three eyes (8.5%) got VA of 0.3–1.5 (Table 4). Visual recovery was much better in eyes with initial VA of LP than that in eyes with initial VA of NLP. In eyes with initial VA of NLP, final vision improved to light perception/ hand movement in 6 eyes (1.7%), counting fingers in 5 eyes (1.4%), 0.02–0.8 in 25 eyes (7.1%).

**Table 2:** Primary management.

**Surgical options**	***n***	**%**
Wound closure	492	78.7
Evisceration	53	8.5
Comprehensive management	46	7.4
Wound closure + cataract removal	23	3.7
No surgery	7	1.1
Other surgeries	4	0.6
Total	625	100

**Table 3:** Secondary intervention.

**Surgical options**	***n***	**%**
Vitrectomy + silicone oil/(+cataract remove)	239	68.3
Evisceration	58	16.6
Vitrectomy/(+cataract remove)	38	10.8
cataract remove/(+IOL implant)	15	4.3
Total	350	100

**Table 4:** Final outcomes of 625 eyes with severe OGIs.

	**LP**		**NLP**	
**Outcomes**	***n***	**%**	***n***	**%**
Loss of follow-up	42	15.4	32	9.1
Evisceration	13	4.8	124	35.2
Atrophy	17	6.2	62	17.6
Atrophy with silicone oil	23	8.4	48	13.6
Silicone oil dependence	31	11.4	49	13.9
NLP-CF	40	14.7	12	3.4
0.01–0.25	60	22	19	5.4
0.3–1.5	47	17.1	6	1.8
Total	273		352	

Of all the 625 severe OGIs, almost half eyes (49.1%) received 2 times of surgery (Table 5). The average was 1.8 times. Seven eyes were followed up with medical treatment alone and 2 eyes received 5 times surgeries. One eye that underwent 5 times surgeries achieved VA of 0.1. Another eye that underwent 5 times surgeries achieved VA of HM.

**Table 5:** Surgery time of 625 eyes with severe OGIs.

**Surgery time**	***n***	**%**
0	7	1.1
1	216	34.6
2	307	49.1
3	80	12.8
4	13	2.1
5	2	0.3
Total	625	100

Of all the 625 severe OGIs, 53 eyes (8.5%) underwent intraocular lens (IOL) related surgery at last. Thirty-seven eyes underwent IOL implant and 16 eyes underwent sclera suture or intrascleral fixation (3).

Of all the eviscerated 137 eyes, 53 eyes (38.4%) were eviscerated at the primary surgery, 68 eyes (49.6%) were eviscerated at the second surgery, 13 eyes (9.5%) were eviscerated at the third surgery and 3 eyes (2.2%) were eviscerated at the fourth surgery.

Of all the 622 cases of severe OGIs, 2 patients were diagnosed sympathetic ophthalmia. One patient was a 50 year-old male patient who sustained an injury to his right eye when riding a bicycle. Ten weeks later he was diagnosed sympathetic ophthalmia. Another patient was a 41 years old male patient who got his right eye hurt by finger and 8 weeks later he was diagnosed sympathetic ophthalmia.

## Discussion

Open globe injury (OGI) remains a significant public health problem both in developed and developing countries due to their outcomes. This type of injury is more commonly seen in underdeveloped and developing countries than in developed countries (1). It is a major and preventable cause of unilateral visual loss.

In spite of medical and technical advancements, severe OGIs result in substantial visual morbidity and lifelong sequelae. Severe OGIs also impose financial burdens on society, company and patients. According to our study, each severe OGI eye need 1.8 times of surgery. Currently, the average medical fee for each severe OGI eye is about 25,000 RMB in Henan Province. In underdeveloped and developing countries, severe OGIs mean disasters to the patients and their families for each patient is the backbone of the family. In our study, the highest incidence of severe adult OGIs was found in the middle-aged group (30–59). The mean age at the time of injury was 47.8 ± 14.1 years.

It seems that patients were younger than those in the study of Fujikawa et al. (4). The mean age of their study populations was 56.7 ± 21.8 years in the LP group and 62.3 ± 21.7 years in the NLP group. This may indirectly reflect the different condition of aging of population of the society. Another interesting difference between our study and Fujikawa et al. (4) is the male to female ratio. In our study, male to female ratio is 81.5% while in the study of Fujikawa et al. (4) it is 66.1%. In other studies, male to female ratio was 73.3–80% (1, 4–12). The highest ratio was found in the study of Supreeyathitikul et al. (13) (88.7%).

In the current study, almost half of the injuries happened at workplace (47.2%) which is similar to the study of Supreeyathitikul et al. (13) (45.8%). In our study, all the severe OGIs cases related to non-wearing of eye protective device. If the patients wear eye protective device, maybe they would not get eyes injured. According to article 54 of <<Safe Production Law of the People's Republic of China>>, the departments in charge of supervising and administering production safety, must strictly check, test or accept the matters concerning production safety, which call for examination and approval, in accordance with the relevant laws and regulations and in conformity with the safety conditions and procedures as required by national standards or trade standards. In fact, Safe Production Law is implemented well in formal company. Safe Production Law seems difficult to enforce when employees work for small unformal company or individuals. Legislation needs improvement to prevent ocular trauma and other traumas.

Of all the 625 severe OGIs, 53 eyes (8.5%) underwent intraocular lens (IOL) related surgery at last. Thirty-seven eyes underwent IOL implant and 16 eyes underwent sclera suture or intrascleral fixation (3). For eyes with combined lens capsular and iris deficiency, glued aniridia IOL and glued IOL with iridoplasty maybe the optimal options (14). Unfortunately, they are unavailable for us.

In our study, 53 eyes were eviscerated at the primary management and 58 eyes were eviscerated at the second intervention. Over 6 months of follow-up, a total of 137 eyes underwent evisceration. The rate of evisceration for severe OGIs in all adult OGIs was 9.2% (137/1,483). It is similar to that of other large series (12, 15, 16). In our study, 124 eyes (35.2%) with initial VA of NLP underwent evisceration. The rate is much lower than that in another small series study (17) in which 21 eyes (84%) were eviscerated in 25 eyes presenting with no light perception (NLP) after open globe injury (OGI). No eyes underwent enucleation in the present study. Zigiotti et al. (18) described a modified standard enucleation.

Before deciding on evisceration/enucleation in severe OGI eyes, reversible causes of vision loss should be excluded including psychological factors (19). Even in situations in which evisceration/enucleation seems inevitable; the ophthalmologist should discuss the possible options with the patient before making a final decision. Primary evisceration/enucleation for severe OGI eyes with NLP in view of risk of sympathetic ophthalmia was a controversial approach. Sympathetic ophthalmia with potential for bilateral blindness is a relative indication for evisceration/enucleation of an injured eye. The use of modern immunosuppressives has also improved treatment and control of sympathetic ophthalmia. In the current study, 2 patients were diagnosed sympathetic ophthalmia after about 2 months later of injury. In consideration of 132 eyes (21.1%) retain some VA and 53 eyes (8.5%) got VA of 0.3–1.5, primary surgical repair should not be abandoned for the risk of sympathetic ophthalmia in eyes with NLP.

In conclusion, this study demonstrated the characteristic, managements, and outcomes of severe OGIs. Severe OGIs are most seen in the young, middle-aged, and male working population and remain a serious public health problem, resulting in significant vision loss or evisceration/enucleation of eyes. Effective preventive measures should be taken for the individuals in these groups. Employers and employees need to be educated on the importance of eye protection. Legislation needs improvement to prevent ocular trauma and other traumas. Finally, we must expand outreach and education to at-risk populations.

## Data Availability Statement

The original contributions presented in the study are included in the article/supplementary material, further inquiries can be directed to the corresponding author.

## Ethics Statement

The studies involving human participants were reviewed and approved by Ethics Committee of Henan Eye Hospital. The patients/participants provided their written informed consent to participate in this study.

## Author Contributions

HC, JH, and XZ organized the database. HC performed the statistical analysis and wrote the first draft of the manuscript. All authors contributed to conception and design of the study, manuscript revision, read, and approved the submitted version.

## Conflict of Interest

The authors declare that the research was conducted in the absence of any commercial or financial relationships that could be construed as a potential conflict of interest.

## Publisher's Note

All claims expressed in this article are solely those of the authors and do not necessarily represent those of their affiliated organizations, or those of the publisher, the editors and the reviewers. Any product that may be evaluated in this article, or claim that may be made by its manufacturer, is not guaranteed or endorsed by the publisher.
